# Mendelian randomization and transcriptomic analysis reveal an inverse causal relationship between Alzheimer’s disease and cancer

**DOI:** 10.1186/s12967-023-04357-3

**Published:** 2023-08-04

**Authors:** Zehua Dong, Mengli Xu, Xu Sun, Xiaosheng Wang

**Affiliations:** 1 Biomedical Informatics Research Lab, School of Basic Medicine and Clinical Pharmacy, China Pharmaceutical University, Nanjing, 211198 China; 2 Cancer Genomics Research Center, School of Basic Medicine and Clinical Pharmacy, China Pharmaceutical University, Nanjing, 211198 China; 3 Big Data Research Institute, China Pharmaceutical University, Nanjing, 211198 China; 4 Department of Pharmacy, Nanjing Luhe People’s Hospital, Nanjing, 211500 China; 5 Department of Pharmacy, Luhe Hospital Affiliated with Yangzhou University Medical College, Nanjing, 211500 China

**Keywords:** Alzheimer’s disease, Cancer, Mendelian randomization, Colocalization analysis, Transcriptome analysis

## Abstract

**Background:**

Alzheimer’s disease (AD) and cancer are common age-related diseases, and epidemiological evidence suggests an inverse relationship between them. However, investigating the potential mechanism underlying their relationship remains insufficient.

**Methods:**

Based on genome-wide association summary statistics for 42,034 AD patients and 609,951 cancer patients from the GWAS Catalog using the two-sample Mendelian randomization (MR) method. Moreover, we utilized two-step MR to identify metabolites mediating between AD and cancer. Furthermore, we employed colocalization analysis to identify genes whose upregulation is a risk factor for AD and demonstrated the genes’ upregulation to be a favorable prognostic factor for cancer by analyzing transcriptomic data for 33 TCGA cancer types.

**Results:**

Two-sample MR analysis revealed a significant causal influence for increased AD risk on reduced cancer risk. Two-step MR analysis identified very low-density lipoprotein (VLDL) as a key mediator of the negative cause-effect relationship between AD and cancer. Colocalization analysis uncovered *PVRIG* upregulation to be a risk factor for AD. Transcriptomic analysis showed that *PVRIG* expression had significant negative correlations with stemness scores, and positive correlations with antitumor immune responses and overall survival in pan-cancer and multiple cancer types.

**Conclusion:**

AD may result in lower cancer risk. VLDL is a significant intermediate variable linking AD with cancer. *PVRIG* abundance is a risk factor for AD but a protective factor for cancer. This study demonstrates a causal influence for AD on cancer and provides potential molecular connections between both diseases.

**Supplementary Information:**

The online version contains supplementary material available at 10.1186/s12967-023-04357-3.

## Introduction

Alzheimer’s disease (AD), characterized by age-related cognitive decline, is the most common neurodegenerative disease to cause dementia and increased risk of mortality in aging populations [1]. Cancer is another age-related disease causing the second most deaths worldwide [2]. Intriguingly, abundant epidemiological evidence suggests an inverse relationship between AD and cancer [3–7]. Furthermore, some studies explored the mechanism underlying the inverse correlation between AD and cancer. For example, it has been reported that immune regulation may links both diseases [8]. The p53 pathway is a potential factor contributing to the correlation between AD and cancer [9]. In addition, a recent study provided biological evidence supporting the inverse correlation between AD and cancer risk by examining Alzheimer’s biomarkers in autopsied brains [10]. Despite these prior studies, the questions on how AD reduce the risk of cancer and vice versa remain unresolved.

Mendelian randomization (MR) is a method of using genetic variants related to biological intermediate of interest to evaluate the cause-effect relationship [11]. This method has been widely utilized to explore the cause-effect relationship between biological or medical variables [12–15]. However, the use of MR to investigate the cause-effect relationship between AD and cancer remains unexplored. In this study, to explore the mechanism of how AD reduce cancer risk, we used two-sample MR [16] to uncover the causal effect of AD on cancer and two-step MR [11] to identify metabolites mediating between AD and cancer. Furthermore, we employed colocalization analysis [17] and transcriptomic analysis to validate the findings by the MR analysis.

## Methods

**Fig. 1:**
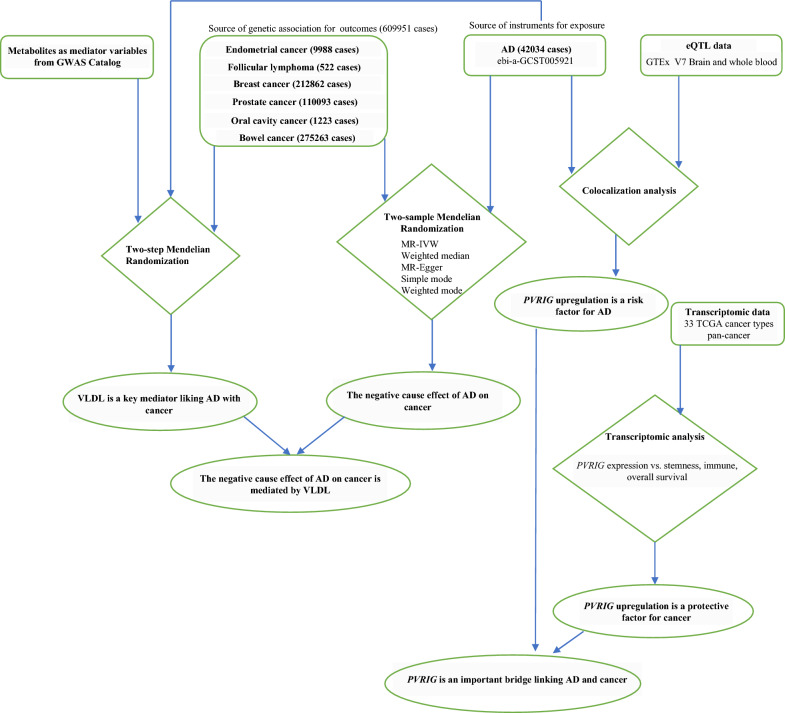
Schematic summary of the study

An illustration of the analytical methods is presented in Fig. 1.

### MR analysis

We utilized two-sample MR analysis to explore the cause-effect relationship between AD and cancer. In the MR analysis, AD was the exposure of interest, cancer was the outcome, and SNPs was instrumental variables. The two-sample MR method was based on the following assumptions: (I) the instrumental variables are strongly associated with AD risk; (II) the instrumental variables influence risk of cancer only through their effect on AD risk; and (III) the instrumental variables are independent of confounders.

We collected genome-wide association summary statistics for 42,034 AD patients and 609,951 cancer patients from the GWAS Catalog (https://www.ebi.ac.uk/gwas/) [18]. Both patient populations were Europeans. The GWAS data for AD (ebi-a-GCST005921 [19]), including 42,034 AD patients and 272,244 controls with 7,746,640 SNPs, was used as the summary association statistics of the exposure (Table 1 and Additional file 1: Table S1). The GWAS data for cancer harbored 13 summary association statistics associated with 6 cancer types [20–24], each of which was used as the summary association statistics of the outcome in turn (Table 1 and Additional file 1: Table S1). We employed five MR methods, including MR Egger, weighted median, inverse-variance weighted (IVW), simple mode, and weighted mode for robust analysis of causality. The causal effects of AD on cancer were determined to be significant based on the criteria of *P* value < 0.05 generated by at least one of the five MR methods (Additional file 1: Table S1). As suggested in previous studies [12, 14, 25], we identified the genetic variants associated with the risk of AD with the threshold *P* < 1 × 10^−5^.

**Table 1:** A summary of the data for Mendelian randomization analysis

	Dataset	# SNPs in GWAS	Cases	Controls	Year
AD	ebi-a-GCST005921	7,746,640	42,034	272,244	2018
Cancer					
Endometrial cancer	ebi-a-GCST006465	9,464,330	8758	46,126	2018
	ebi-a-GCST006466	8,947,630	1230	35,447	2018
Follicular lymphoma	finn-b-CD2_FOLLICULAR_LYMPHOMA_EXALLC	16,380,337	522	180,756	2021
Breast cancer	ieu-a-1126	10,680,257	122,977	105,974	2017
	ieu-a-1132	10,680,257	38,197	45,494	2017
	ukb-b-12227	9,851,867	16,586	345,223	2018
	ukb-b-13584	9,851,867	35,102	388,356	2018
Prostate cancer	ieu-b-85	20,346,368	79,148	61,106	2018
	ukb-b-7773	9,851,867	30,945	368,725	2018
Oral cavity cancer	ieu-b-94	7,510,833	1223	2928	2016
Bowel cancer	ukb-a-296	10,894,596	107,072	224,939	2017
	ukb-b-7748	9,851,867	22,028	401,107	2018
	ukb-b-17001	9,851,867	146,163	309,096	2018

In addition, we utilized two-step MR analysis to identify mediator variables of metabolites mediating the cause-effect relationship between AD and cancer. The GWAS data for metabolites were also obtained from the GWAS Catalog (https://www.ebi.ac.uk/gwas/) [18] (Additional file 2: Table S2). In the two-step MR analysis, β0 − β1 × β2 was utilized as the direct effect of exposure on outcome [26], where β0 measures the causal effect of the exposure on the outcome, β1 the causal effect of the exposure to the mediator, β2 the causal effect of the mediator to the outcome, and β1 × β2 represents the mediating effect from the exposure to the outcome.

We assessed the directional pleiotropy based on the intercept obtained from the MR-Egger analysis [27]. The R package “TwoSampleMR” and the web tool MRbase (http://app.mrbase.org/) were used for two-sample MR and two-step MR analysis, respectively.

### Colocalization analysis

We employed colocalization analysis to identify target genes for AD. The colocalization analysis integrated information from all eQTL SNPs, including those in cis and trans [28] by combining eQTL data for multiple tissues and GWAS data. When GWAS signaling and eQTL colocalization are detected, the GWAS loci may influence the expression phenotypes of target genes [29]. We identified target genes at risk loci for AD based on the value of the log Bayes Factor (LBF). A larger LBF represents a stronger association between loci and genes [30]. In the colocalization analysis, we employed the GWAS data for AD “ebi-a-GCST005921” and the expression quantitative trait loci (eQTL) data for whole blood and brain from the Genotype-Tissue Expression (GTEx) (https://www.gtexportal.org/home/index.html) (Additional file 3: Table S3). Since this is an AD-associated colocalization analysis, we selected the eQTL data from GTEx associated with blood and brain tissues, including whole blood, brain cerebellum, brain caudate basal ganglia, brain cortex, brain nucleus accumbens basal ganglia, brain cerebellar hemisphere, brain frontal cortex BA9, brain putamen basal ganglia, brain hippocampus, brain anterior cingulate ganlia, brain hypothalamus, brain amygdala, brain spinal cord cervical and brain substantia nigra. We implemented the colocalization analysis with the R package “coloc” [31] and the web tool Sherlock (http://sherlock.ucsf.edu/submit.html).

### Transcriptomic analysis

Based on transcriptomic data (RSEM-normalized RNA-Seq gene expression profiles) from TCGA (https://portal.gdc.cancer.gov/), we analyzed the correlations between the expression of an AD risk gene (*PVRIG*) and molecular and clinical features in 33 cancer types and pan-cancer. The molecular and clinical features included stemness, immune, and overall survival (OS). The 33 cancer types included adrenocortical carcinoma (ACC), bladder urothelial carcinoma (BLCA), breast invasive carcinoma (BRCA), cervical squamous-cell carcinoma (CESC), cholangiocarcinoma (CHOL), colon adenocarcinoma (COAD), lymphoid neoplasm diffuse large B-cell lymphoma (DLBC), esophageal carcinoma (ESCA), glioblastoma multiforme (GBM), head and neck squamous cell carcinoma (HNSC), kidney chromophobe (KICH), kidney renal clear cell carcinoma (KIRC), kidney renal papillary cell carcinoma (KIRP), acute myeloid leukemia (LAML), brain lower grade glioma (LGG), liver hepatocellular carcinoma (LIHC), lung adenocarcinoma (LUAD), lung squamous cell carcinoma (LUSC), mesothelioma (MESO), ovarian carcinoma (OV), and pancreatic adenocarcinoma (PAAD), pheochromocytoma and paraganglioma (PCPG), prostate adenocarcinoma (PRAD), rectum adenocarcinoma (READ), sarcoma (SARC), skin cutaneous melanoma (SKCM), stomach adenocarcinoma (STAD), testicular germ cell tumors (TGCT), thyroid carcinoma (THCA), thymoma (THYM), uterine corpus endometrial carcinoma (UCEC), uterine carcinosarcoma (UCS), and uveal melanoma (UVM).

We utilized the single-sample gene set enrichment analysis (ssGSEA) [32, 33] to evaluate the enrichment levels of stemness and immune signatures based on the expression profiles of their marker genes. The marker genes for stemness [34] and immune signatures [35, 36] are shown in Additional file 4: Table S4. We employed the Pearson or Spearman method to evaluate the correlation between two groups of data. We compared OS time between cancer patients with higher gene expression (> median) and those with lower gene expression (< median) by the Kaplan–Meier estimator [37]. The log-rank test *P* < 0.05 indicated the significance of survival time differences. We implemented survival analysis with the function “survfit ()” in the R package “survival.”

In addition, we used the Benjamini–Hochberg method [38] to calculate the false discovery rate (FDR) for adjusting for *P* values in multiple tests.

## Results

### MR analysis reveals a negative cause-effect relationship between AD and cancer

In the two-sample MR analysis, SNPs was taken as the instrumental variable, AD as the exposure of interest and cancer as the outcome. The GWAS data for AD (ebi-a-GCST005921 [19]) was used as the summary association statistics of the exposure and each of the 13 GWAS data for cancer the summary association statistics of the outcome (Table 1 and Additional file 1: Table S1). The GWAS data for endometrial cancer “ebi-a-GCST006465” [20] included 8,758 cancer patients and 46,126 controls with 9,464,330 SNPs. MR analysis demonstrated a significant causal influence for increased AD risk on reduced risk of endometrial cancer (*P*_MR-Egger_ = 0.025, *P*_weighted-median_ = 0.004, *P*_IVW_ = 0.014, *P*_simple-mode_ = 0.256 and *P*_weighted-mode_ = 0.015; Table 2). The heterogeneity assessment showed little evidence of heterogeneity for the association (Cochran’s Q_MR-Egger_ = 6.29 and *P* = 0.61, Q_IVW_ = 8.23 and *P* = 0.51; Table 2). In addition, horizontal pleiotropy analysis showed little evidence of pleiotropy for the association (*P* = 0.097; Table 2).

**Table 2:** Two-sample MR results of AD as the exposure and cancer as the outcome

Endometrial cancer (ebi-a-GCST006465)	Method	nsnp	β	se	pval
Results	MR Egger	10	− 0.1507	0.0547	0.0249
	Weighted median	10	− 0.1279	0.0441	0.0037
	IVW	10	− 0.0991	0.0402	0.0138
	Simple mode	10	− 0.1297	0.1070	0.2561
	Weighted mode	10	− 0.1297	0.0433	0.0150

	Method	Q	Q_df	Q_pval	
Heterogeneity test	MR Egger	6.2937	8	0.6144	
	IVW	8.2313	9	0.5110	

	egger_intercept	se	pval		
Test for directional horizontal pleiotropy	0.0126	0.009	0.2014		

Breast cancer (ukb-b-13584)	Method	nsnp	β	se	pval
Results	MR Egger	10	− 0.0039	0.0022	0.1129
	Weighted median	10	− 0.0034	0.0014	0.0144
	IVW	10	− 0.0029	0.0016	0.0604
	Simple mode	10	− 0.0012	0.0032	0.7121
	Weighted mode	10	− 0.0034	0.0013	0.0333

	Method	Q	Q_df	Q_pval	
Heterogeneity test	MR Egger	12.9930	8	0.1121	
	IVW	13.6812	9	0.1341	

	egger_intercept	se	pval		
Test for directional horizontal pleiotropy	0.0002	0.0004	0.5333		

Bowel cancer (ukb-b-17001)	Method	nsnp	β	se	pval
Results	MR Egger	10	− 0.0053	0.0028	0.0988
	Weighted median	10	− 0.0061	0.0024	0.0096
	IVW	10	− 0.0060	0.0021	0.0036
	Simple mode	10	− 0.0085	0.0051	0.1336
	Weighted mode	10	− 0.0062	0.0024	0.0302

	Method	Q	Q_df	Q_pval	
Heterogeneity test	MR Egger	4.6927	8	0.7899	
	IVW	4.8647	9	0.8459	

	egger_intercept	se	pval		
Test for directional horizontal pleiotropy	− 0.0002	0.0005	0.6893		

Breast cancer is the most common cancer in women and also the most common cancer overall [39]. The GWAS data for breast cancer “ukb-b-13584” [20] included 35,102 cancer patients and 388,356 controls with 9,851,867 SNPs. MR analysis showed a significant, negative, causal influence for AD risk on the risk of breast cancer (*P*_MR-Egger_ = 0.113, *P*_weighted-median_ = 0.014, *P*_IVW_ = 0.060, *P*_simple-mode_ = 0.712 and *P*_weighted-mode_ = 0.033; Table 2). This analysis showed no significant heterogeneity (Q_MR-Egger_ = 12.99 and *P* = 0.11, Q_IVW_ = 13.68 and *P* = 0.13; Table 2) or horizontal pleiotropy for the association (*P* = 0.53; Table 2).

Bowel cancer, also known as colorectal cancer, is the third most common cancer worldwide [39]. The GWAS data for bowel cancer “ukb-b-17001” [20] included 146,163 cancer patients and 309,096 controls with 9,851,867 SNPs. As well, MR analysis suggested a causal influence for increased AD risk on reduced risk of bowel cancer (*P*_MR-Egger_ = 0.099, *P*_weighted-median_ = 0.010, *P*_IVW_ = 0.004, *P*_simple-mode_ = 0.134 and *P*_weighted-mode_ = 0.030; Table 2). This analysis showed little evidence of heterogeneity (Q_MR-Egger_ = 4.69 and *P* = 0.79, Q_IVW_ = 4.86 and *P* = 0.85; Table 2) or horizontal pleiotropy for the association (*P* = 0.69; Table 2).

MR analysis also revealed a negative causal influence for AD risk on risk of other cancers, such as prostate cancer, follicular lymphoma, and oral cavity cancer (Additional file 1: Table S1). Inversely, when cancer was taken as the exposure of interest and AD as the outcome, MR analysis showed no significant causal effect of cancer on AD.

To explore the mechanism underlying the causal effects of AD on cancer, we performed two-step MR analysis with metabolites as mediator variables. When the GWAS data for AD (ebi-a-GCST005921 [19]) as the exposure of interest and the GWAS data for endometrial cancer “ebi-a-GCST006465” [20] as the outcome, we found very-low-density lipoprotein (VLDL) to be a significant intermediate variable linking AD with cancer (IVW method) (Table 3). That is, a positive causal effect of AD on VLDL (AD as the exposure of interest and VLDL as the outcome) (*P*_IVW_ < 0.01; β > 0) and a negative causal effect of VLDL on cancer (VLDL as the exposure of interest and cancer as the outcome) (*P*_IVW_ < 0.05; β < 0) were uncovered. Likewise, MR analysis demonstrated VLDL to be a significant intermediate variable linking AD with cancer in analyzing the GWAS data for other cancer cohorts (Additional file 2: Table S2).

**Table 3:** Two-step MR results of VLDL as a mediator variable for AD and cancer

	VLDL	nsnp	β	se	pval	Q	Q_df	Q_pval
AD as the exposure and VLDL as the outcome	L.VLDL.C	10	0.1109	0.0298	0.0002	18.0300	9	0.0348
	L.VLDL.CE	10	0.0969	0.0298	0.0012	16.9641	9	0.0493
	L.VLDL.PL	10	0.1416	0.0293	0.0000	17.3709	9	0.0432
	S.VLDL.FC	10	0.2240	0.0332	0.0000	22.7703	9	0.0067
	S.VLDL.L	10	0.1986	0.0319	0.0000	19.7753	9	0.0194
	S.VLDL.P	10	0.1840	0.0321	0.0000	19.9873	9	0.0180
	S.VLDL.PL	10	0.1837	0.0295	0.0000	18.0111	9	0.0350
VLDL as the exposure and endometrial cancer as the outcome	L.VLDL.C	9	− 0.1487	0.0678	0.0282	5.9211	8	0.6561
	L.VLDL.CE	11	− 0.1503	0.0696	0.0308	12.4403	10	0.2565
	L.VLDL.PL	9	− 0.1946	0.0768	0.0112	9.8292	8	0.2773
	S.VLDL.FC	10	− 0.1523	0.0649	0.0188	9.5730	9	0.3862
	S.VLDL.L	11	− 0.1282	0.0695	0.0650	13.3105	10	0.2070
	S.VLDL.P	12	− 0.1420	0.0759	0.0615	13.3105	10	0.2070
	S.VLDL.PL	10	− 0.1517	0.0625	0.0153	9.5537	9	0.3879

		Mediating effect	Direct effect					
AD as the exposure, endometrial cancer as the outcome, and VLDL as the mediator	L.VLDL.C	− 0.0165	− 0.0826					
	L.VLDL.CE	− 0.0146	− 0.0845					
	L.VLDL.PL	− 0.0276	− 0.0715					
	S.VLDL.FC	− 0.0341	− 0.0650					
	S.VLDL.L	− 0.0255	− 0.0736					
	S.VLDL.P	− 0.0261	− 0.0730					
	S.VLDL.PL	− 0.0279	− 0.0712					

Prefix—*S* small size, *L* large size

Suffix—*C* total cholesterol, *CE* cholesterol esters, *PL* phospholipids, *FC* free cholesterol, *L* total lipids, *P* concentration of particles

Taken together, MR analysis reveals a significant, negative causal effect of AD on cancer and VLDL acting as an intermediate variable mediating the relationship between AD and cancer.

### Identification of target genes and risk loci for AD by colocalization analysis

Expression quantitative trait loci (eQTL) are genetic variants associated with gene expression phenotypes [40]. Since eQTL data are tissue-specific, we only used eQTL data for whole blood and brain for AD-associated colocalization analysis. When using the eQTL data “GTEx V7 Brain nucleus accumbens basal ganglia” and the GWAS data for AD “ebi-a-GCST005921” for colocalization analysis, certain genes whose expression showed significant positive associations with risk loci for AD were identified (*P* < 0.05, LBF > 0; Table 4). These genes included *PVRIG* (*P* < 0.001, LBF = 7.39), *KAT8* (*P* < 0.001, LBF = 7.27), and *STAG3* (*P* < 0.001, LBF = 6.73) (Table 4). Among these genes, *PVRIG* was commonly identified by analyzing the eQTL data for whole blood and different brain regions (Additional file 3: Table S3). It suggests that elevated expression of *PVRIG* is a risk factor for AD.

**Table 4:** Results by colocalization analysis of eQTL data for Brain nucleus accumbens basal ganglia and GWAS data for AD

Gene	SNP	Location	Proximity	eQTL pval
*PVRIG*	rs1061230	chr7:99645201	cis	1.82e−08
*KAT8*	rs10871454	chr16:30955580	cis	3.75e−06
*PRSS36*	rs1060506	chr16:31040950	cis	8.60e−14
*STAG3*	rs1138417	chr7:99645082	cis	1.24e−11
*SIGLEC11*	rs10405621	chr19:55147163	cis	3.19e−06
*CEBPZ-AS1*	rs11124575	chr2:37353939	cis	6.36e−06
*NUDT2*	rs10814087	chr9:34255763	cis	6.32e−12
*AP4M1*	rs10281368	chr7:99493833	cis	8.68e−07
*CYP2D7P*	rs1033460	chr22:40949252	cis	9.91e−11

GWAS pval	LBF	Nearby genes	Nearby TF	p-value
3.50e−06	7.39	*STAG3*, *GPC2*, more…	No	5.72E−06
1.46e−06	7.27	*STX1B*, *PRSS53*, more…	Yes	5.72E−06
1.18e−05	7.13	*KAT8*, *PRSS53*, more…	Yes	5.72E−06
3.38e−06	6.73	*STAG3*, *GPC2*, more…	No	1.14E−05
9.35e−05	5.76	*MIR4751*, *NUP62*, more…	Yes	5.72E−05
8.37e−05	4.89	*CEBPZ*, *NDUFAF7*, more…	Yes	1.37E−04
6.47e−04	3.54	*KIF24*, *UBAP1*	No	6.63E−04
3.94e−04	3.53	*MCM7*, *ZNF3*, *TAF6*, more…	Yes	6.75E−04
7.34e−04	3.33	*TCF20*, *OGFRP1*	Yes	7.89E−04

### *PVRIG* upregulation is associated with favorable outcomes in cancer

**Fig. 2:**
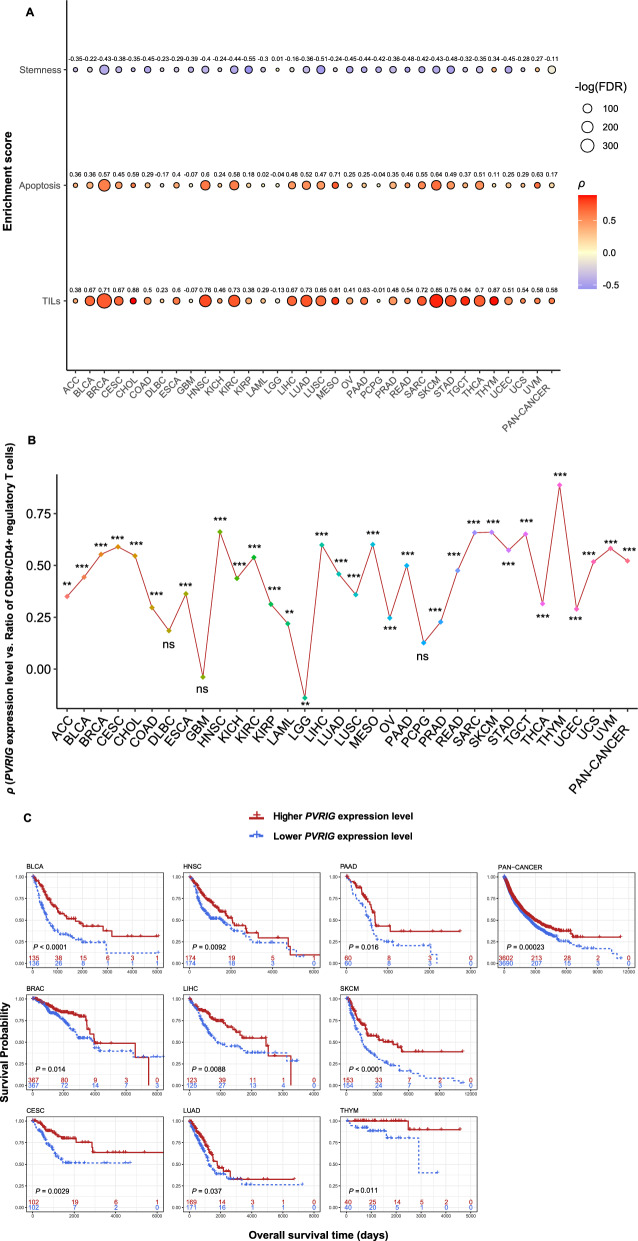
Transcriptomics analysis reveals *PVRIG* upregulation to be associated with favorable outcomes in cancer. Correlations between *PVRIG* expression levels and stemness scores, apoptosis pathway’s enrichment scores, enrichment scores of tumor-infiltrating lymphocytes (TILs) (**A**), and ratios of CD8+/CD4+ regulatory T cells (**B**) in pan-cancer and in 33 individual cancer types. **C** Kaplan–Meier survival curves showing better overall survival in higher-*PVRIG*-expression-level (upper third) than in lower-*PVRIG*-expression-level (bottom third) cancer patients in pan-cancer and in nine individual cancer types. The Spearman’s correlation coefficients (*ρ*) and adjusted *P* values (FDR) are shown in **A**; the Pearson’s correlation coefficients (*r*) and FDR are shown in **B**; and the log-rank test *P* values are shown in **C**. *FDR < 0.05; **FDR < 0.01; ***FDR < 0.001; ^ns^FDR ≥ 0.05

To explore the role of *PVRIG* in cancer, we analyzed the associations between *PVRIG* expression and various molecular and clinical features in 33 TCGA cancer types, including stemness, immune, and survival prognosis. Stem cell-like characteristics in a fraction of cancer cells may confer cancer progression and treatment resistance [35]. Notably, *PVRIG* displayed significant negative expression correlations with stemness scores in pan-cancer and in 30 individual cancer types (Spearman correlation, FDR < 0.05) (Fig. 2A). *PVRIG* expression was significantly and positively correlated with the apoptosis pathway’s enrichment scores in pan-cancer and in 26 individual cancer types (FDR < 0.05) (Fig. 2A). In pan-cancer and in 30 individual cancer types, *PVRIG* expression was positively correlated with the enrichment scores of TILs (FDR < 0.05) (Fig. 2A). Moreover, *PVRIG* had a significant positive expression correlation with the ratios of CD8+/CD4+ regulatory T cells in pan-cancer and in 29 individual cancer types (Pearson correlation, FDR < 0.05) (Fig. 2B). These results collectively suggest that elevated expression of *PVRIG* is associated with active antitumor immune responses. Furthermore, in pan-cancer and in nine common cancer types (BLCA, BRCA, CESC, HNSC, LIHC, LUAD, PAAD, SKCM and THYM), increased expression of *PVRIG* was correlated with better OS (*P* < 0.05) (Fig. 2C). Taken together, these data suggest that *PVRIG* is a tumor suppressor gene in cancer.

## Discussion

For the first time, we used the two-sample MR method to explore the causal effect of AD on cancer. This analysis supports a significant causal influence for increased AD risk on reduced cancer risk, consistent with previous reports of the inverse relationship between AD and cancer [3–7]. Furthermore, we employed two-step MR to identify potential mediators of metabolites linking AD with cancer. We found VLDL to be a key mediator of the negative cause-effect relationship between AD and cancer. Finally, we uncovered *PVRIG* upregulation to be a risk factor for AD by colocalization analysis, while *PVRIG* likely plays a role in tumor suppression by transcriptomic analysis, as evidenced by that *PVRIG* expression had significant negative correlations with stemness scores, and positive correlations with antitumor immune responses and overall survival. It suggests that *PVRIG* could be an important bridge linking AD and cancer.

Our results are in agreement with prior studies. For example, a recent study revealed elevated levels of VLDL in AD patients [41]. In contrast, another study demonstrated a significant reduction of VLDL levels in cancer patients [42]. Guen et al. [43] showed that *PVRIG* had the strongest eQTL association at the *PILRA* locus, a risk locus for AD. a recent study [44] showed that tumors highly expressing *PVRIG* were characterized by high levels of TILs, strong antitumor immune responses and favorable survival, in line with our results.

Interestingly, in a few cancer types, such as LGG, GBM, and DLBC, the association between *PVRIG* expression and the molecular features showed different results with most of the other cancer types (Fig. 2A). It indicates that the relationship between AD and cancer risk is positive in the few cancer types. This indication is supported by previous reports. For example, a previous epidemiological investigation revealed a significant positive association between AD mortality and malignant brain tumor mortality in people aged 65 and older in the US [45]. In addition, previous studies showed that TREM2 (Triggering Receptor Expressed On Myeloid Cells 2) acts as a risk factor for both AD and brain tumors [46, 47]. However, to date there are very few reports on the relationship between AD and DLBC risk that would be an interesting direction for investigation.

Our study may provide molecular insights into why AD patients are not susceptible to cancer, a conclusion established by epidemiological observations. Our findings suggest that the immune system may be an important factor responsible for the inverse relationship between AD and cancer risk. However, there are several limitations in this study. First, we did not perform experimental verification of the tumor suppressive effect of *PVRIG*. Second, the role of VLDL as a mediator linking AD with cancer remains further proved by experimental and clinical data. Finally, it is worthy of exploring whether immunity is a key factor mediating the relationship between AD and cancer, since immune system has been shown to have associations with both disease [48, 49].

## Conclusion

AD may result in lower cancer risk. VLDL is a significant intermediate variable linking AD with cancer. *PVRIG* abundance is a risk factor for AD but a protective factor for cancer. This study demonstrates a causal influence for AD on cancer and provides potential molecular connections between both diseases.

### Supplementary Information

**Additional file 1: Table S1.** Two-sample MR results of AD as the exposure and 13 cancer cohorts as the outcome and detailed description of related data.

**Additional file 2: Table S2.** Two-step MR results of VLDL as a mediator variable for AD and 13 cancer cohorts and detailed description of VLDL-related data.

**Additional file 3: Table S3.** Results by colocalization analysis of eQTL data for whole blood and 13 brain regions and GWAS data for AD.

**Additional file 4: Table S4.** Marker genes of stemness, immune, and apoptosis signatures or pathways.

## Data Availability

All data associated with this study are available within the paper and its Additional files.
